# The effects of the DDS-1 strain of lactobacillus on symptomatic relief for lactose intolerance - a randomized, double-blind, placebo-controlled, crossover clinical trial

**DOI:** 10.1186/s12937-016-0172-y

**Published:** 2016-05-20

**Authors:** Michael N. Pakdaman, Jay K. Udani, Jhanna Pamela Molina, Michael Shahani

**Affiliations:** 1 Pakdaman Consulting, 22287 Mulholland Hwy #269, Calabasas, CA 91302 USA; 2 Northridge Hospital Integrative Medicine Program, 18300 Roscoe Blvd, Northridge, CA 91328 USA; 3 Nebraska Cultures, 45 Quail Ct #206, Walnut Creek, CA 94596 USA

**Keywords:** Lactobacillus acidophilus, Lactose intolerance, Lactose malabsorption, Probiotics, Diarrhea, Vomiting

## Abstract

**Background:**

Lactose intolerance is a form of lactose maldigestion where individuals experience symptoms such as diarrhea, abdominal cramping, flatulence, vomiting and bowel sounds following lactose consumption. *Lactobacillus acidophilus* is a species of bacteria known for its sugar fermenting properties. Preclinical studies have found that *Lactobacillus acidophilus* supplementation may assist in breaking down lactose; however, no human clinical trials exist evaluating its efficacy in alleviating symptoms related to lactose intolerance.

**Objective:**

The aim of this randomized, double-blind, placebo-controlled, crossover study was to evaluate the effect of a proprietary strain of *Lactobacillus acidophilus* on relieving discomfort related to lactose intolerance.

**Methods:**

The study enrolled healthy volunteers between 18 and 75 years of age who complained of lactose intolerance. Screening visits included a lactose challenge visit to confirm eligibility based on a score of 10 or higher on subjective assessment of the following symptoms after lactose challenge: diarrhea, abdominal cramping, vomiting, audible bowel sounds, flatulence, and overall symptoms. Qualified subjects participated in a 2-arm crossover design, with each arm consisting of 4 weeks of intervention of either active or placebo product, with a 2-week washout period during crossover. The study product consisted of the DDS-1 strain of *Lactobacillus acidophilus* (Nebraska Cultures, Walnut Creek, California). The placebo was formulated from maltodextrin. Study participants were instructed to take the product once daily for 4 weeks. Data collected included subjective symptom scores related to lactose intolerance.

**Results:**

Longitudinal comparison between the DDS-1 group and placebo group demonstrated statistically significant reductions in abdominal symptom scores during the 6-h Lactose Challenge at week 4 for diarrhea (*p* = 0.033), abdominal cramping (*p* = 0.012), vomiting (*p* = 0.0002), and overall symptom score (*p* = 0.037). No adverse events were reported.

**Conclusions:**

The present study has found that this unique DDS-1 strain of *Lactobacillus acidophilus*, manufactured by Nebraska Cultures, is safe to consume and improves abdominal symptom scores compared to placebo with respect to diarrhea, cramping, and vomiting during an acute lactose challenge.

## Introduction

Lactose maldigestion is a common condition affecting up to 75 % of the world's population [1]. It results from a normal physiologic decline in the activity of the brush-border enzyme lactase, or beta-d-galactosidase [1, 2]. The lactase enzyme is responsible for cleavage of lactose, a disaccharide carbohydrate consisting of glucose and galactose and commonly found in mammalian milk [3]. When the amount of ingested lactose exceeds the hydrolytic capacity of lactase in the intestine, undigested lactose is transported to the large intestine where it is fermented by the bacterial microflora, producing organic acids, carbon dioxide, and hydrogen. These byproducts, along with the large amount of water osmotically drawn into the intestine, lead to the commonly known symptoms of abdominal pain, bloating, cramps, and flatulence [4, 5]. Audible bowel sounds have also been described as a common complaint among lactose maldigesters [6–8]. Lactase expression reaches its peak at birth in preparation for breastfeeding and begins to wean from that point until it reaches undetectable levels [3]. Gastrointestinal pathology such as infection, inflammatory bowel disease, and abdominal surgery can hasten this process [5].

It is important to distinguish lactase deficiency from lactose malabsorption and lactose intolerance. *Lactase deficiency* involves the natural physiologic reduction in brush-border lactase activity that initiates after birth [5]. *Lactose malabsorption* is the process whereby lactose is not absorbed in the intestine. *Lactose intolerance* is defined as the clinical syndrome of hypolactasia and lactase maldigestion resulting from the physiologic process of lactose maldigestion [9]. The extent to which undigested lactose causes the lactose intolerance symptoms depends on a number of factors, including amount of lactose ingested, small-intestinal lactase activity, gastric emptying rate, transit time, and gastrointestinal microflora composition [3, 10]. Lactose maldigesters can consume 0.5–7 g of lactose, which is equivalent to approximately 3 oz of milk, without experiencing symptoms of intolerance [11]. Lactose maldigestion, therefore, does not necessarily lead to lactose intolerance.

While the gold standard for diagnosis of lactase deficiency is through gastrointestinal mucosal biopsy, lactose maldigestion in humans is often assessed using the hydrogen breath test (HBT) [12, 13]. The HBT measures the concentration of exhaled hydrogen after consumption of lactose, specifically defined as > 20 ppm in one breath after consumption of 50 g lactose. The increase in exhaled hydrogen results from the release of hydrogen-containing methane byproducts during bacterial ingestion of lactose [14]. Lactose intolerance, the clinical manifestation of lactose malabsorption, is a subjective phenomenon commonly assessed using self-reported symptom assessment scales [14].

Conventional management of lactose intolerance primarily involves curtailment of dairy product consumption [15, 16]. However, as dairy products provide an excellent source of calcium, potassium, vitamin D, B vitamins and high quality protein, avoidance of these foods may increase the risk for bone fracture, osteoporosis and other adverse health effects [17, 18]. Another commonly used treatment option involves supplementation with the lactase enzyme [19, 20]. Recent trends aim to alter the natural intestinal flora to create an environment more conducive to lactose breakdown and absorption through the use of probiotic supplements [15, 21]. Alteration of colonic bacteria is thought to increase the intra-intestinal lactase activity and thus reduce the effects of fermentation products [22, 23]. A number of studies have demonstrated that cultured yogurt possess considerable enzyme activity primarily due to the lactase produced by lactic acid bacteria (LAB) such as Lactobacillus acidophilus and Lactobacillus bulgaricus [24–27]. This is beneficial to individuals suffering from lactose intolerance because of reduced lactose and lactase produced [4]. Numerous research studies have also shown that LAB encourages the growth of proteolic enzymes and lipases involved in the breakdown of protein, fat, and carbohydrates [28–31]. L. acidophilus has also been shown to carry antimicrobial properties [32–36] as well as anticarcinogenic and immunological properties [37–42].

The DDS-1 strain of *Lactobacillus acidophilus*, discovered in 1959 by Dr. Khem Shahani at the University of Nebraska, is a unique strain of *L. acidophilus* on deposit with the FDA Agricultural Research Service (ARS) with the catalog number B-3208. It is currently manufactured by Nebraska Cultures, Inc. A recent study demonstrated the DDS-1 strain of *L. acidophilus* to be superior to other strains of lactobacillus in the ability to establish in the human gastrointestinal (GI) tract [43]. This present study evaluates the effect of *L. acidophilus* DDS-1 in alleviating the symptoms associated with lactose intolerance.

## Methods

### Investigational product

The investigational product for this study was the DDS-1 strain of *Lactobacillus acidophilus* (Nebraska Cultures, Inc., Walnut Creek, California). The placebo was dispensed in an identical capsule formulated from maltodextrin. The sponsor provided both the DDS-1 study product and placebo product. Blinding was ensured with the use of identical bottles, outer packaging, labeling and color for the investigation products. Study participants were instructed to take one capsule with food once daily (every morning) for 4 weeks. The capsules with DDS-1 *L. acidophilus* contained not less than 10 billion (1 X 10^10^) CFU per dose. As the product and placebo are not classified as pharmaceutical drugs and the study does not aim to satisfy claims to diagnose, treat, or prevent a disease process, it does not fall under the category of investigational drug. Thus, Food and Drug Administration review is not required for execution of this clinical trial.

### Study participants

Study subjects were recruited via online advertising and were phone screened prior to scheduling an in-clinic screening visit. Inclusion criteria included age between 18 and 75 years, body mass index (BMI) of between 18 and 35 kg/m^2^, score of 10 or higher on 6-h symptom score following lactose challenge, and agreement to stop consumption of probiotic products or lactose digestion aids two weeks prior to the study. Females of child bearing potential agreed to use appropriate birth control methods during the entire study period. Subjects with congenital lactose deficiency, any significant GI condition including ulcerative colitis, Crohn’s disease, or diarrhea as well as any clinically significant medical conditions (e.g., surgery within the past six months, history of seizure) were excluded from the study. The full list of inclusion and exclusion criteria are detailed in Table 1.

**Table 1:** Inclusion and exclusion criteria

Inclusion criteria	Exclusion criteria
• Healthy volunteers ≥ 30 and ≤ 75 years of age. • Body mass index (BMI) ≥ 18 and ≤ 35 kg/m2. • Lactose Challenge Test 6-hour Symptom Score (SSC) >10 • Judged by the Investigator to be in good health. • Females of child bearing potential must agree to use appropriate birth control methods during the entire study period. • Agree not to initiate any new exercise or diet programs during the entire study period. • Agree to halt consumption of probiotic products or lactose digestion aids two weeks prior to study. • Agree not to change their current diet or exercise program during the entire study period. • Understands the study procedures and signs forms providing informed consent to participate in the study and authorization for release of relevant protected health information to the study investigator.	• Subjects with congenital lactose deficiency. • Any significant GI condition that would potentially interfere with the evaluation of the study product including but not limited to: o Inflammatory bowel disease (Ulcerative Colitis or Crohn’s Disease) o Frequent diarrhea o Surgery for weight loss (e.g., gastric bypass or lap band) o History of gastrointestinal perforation. o History of gastroparesis. • Recent (within two weeks of Visit 1) episode of acute gastrointestinal illness such as nausea, vomiting, or diarrhea. • Consumption of antibiotics and/or laxatives within the three months prior to the study • History of immunocompromise or auto-immune disorder. • Use of any immunosuppressive drugs in the last 12 months (including steroids or biologics) • Active infection requiring antibiotics, anti-viral medication, or hospitalization • Subjects with known sensitivities to the ingredients in the study product • Subjects who are pregnant or lactating • Subjects with a history of seizure • Subjects on anticoagulation therapy • Subjects with known alcohol abuse or recreational drug abuse • Subjects with brain and/or spinal cord injury • Untreated or unstable hypothyroidism • Subjects with any cancer in the last 5 years (except non-melanoma skin cancer) • Surgery within the last 6 months • Any clinically significant burn within the last 6 months • Abnormal physical examination • Subjects unable to understand or follow the study protocol

The lactose challenge was used to determine eligibility during screening; subjects were included if they had a symptom score of 10 or more during an in-clinic lactose challenge test where lactose was dissolved in water and consumed. Lactose at 25 g per dose, which is equivalent to 480 ml of milk, was used in this study instead of the usual 50 g per dose for the lactose HBT [44] because it more closely reflects the average amount of milk consumed by the population [7].

### Study Design

This study was a randomized, double-blind, placebo-controlled, crossover clinical trial. Group allocation was placed in individually numbered envelopes to maintain blinding of all individuals. The subjects, as well as the clinical staff, data management staff, and statistical analysis staff were unaware of which study group each participant was assigned. Medicus Research was the contract research organization (CRO) for this study. Institutional review board (IRB) approval was received on January 24, 2013 by the IntegReview Ethical Review Board (Austin, TX) prior to the initiation of any study- related activities.

A statistical power calculation was performed on data from the Ojetti et al. study [20]. Using the Wilcoxon Mann-Whitney test, with a power of 0.80 and an alpha of 0.05, the required sample size per group was 20. Therefore, a decision was made to aim to include at least 30 subjects in a crossover fashion (30 per group) to ensure that the study was adequately powered.

The study duration was twelve weeks with a total of 8 visits for each subject. The initial screening visit (visit 1) was performed two weeks prior to the baseline visit. During screening, study participants underwent a detailed and thorough informed consent process. Study procedures only commenced after the study participant agreed that he/she understood all details of the consent documentation and the specific details of the study as outlined in the consent document. After the consent was signed, study-related procedures were performed, including review of all inclusion and exclusion criteria. The screening process also included a detailed medical history, review of prior and concomitant medications, physical examination, vital signs, anthropometric measures, and urine pregnancy testing. Subjects were dispensed standardized foods, which were to be consumed within 48-h prior to the next visit, and were instructed to complete a gut health diary and GI symptom questionnaire. Subjects returned to the clinic for visit 1.5 having fasted for 10-h and having only consumed the standardized foods, ensuring controlled intake of sugar, carbohydrate, fiber, and dairy products for 48-h prior to the visit. The purpose of labeling the visit 1.5 is because the subjects were still in the screening phase of the study and in order to conform with common standards of clinical trials, the randomization visit is considered "visit 2." The reason that visit 1.5 procedures cannot be performed during screening is that the subject needs to be provided with the standardized foods and must sign the informed consent document prior to being asked to fast for 10-h. During visit 1.5, subjects underwent the lactose challenge test, where they ingested 25 g of lactose dissolved in water. Subjects were administered a validated 6-h symptom score (SSC) questionnaire consisting of six abdominal-related items: diarrhea, abdominal cramping, vomiting, audible bowel sounds, flatulence, and overall symptoms hourly for 6-h. Subjects also performed the HBT 10-min prior to lactose consumption and at Time = 60-min, 120-min, and 180-min. Prior to leaving the clinic, they were dispensed standardized foods, diaries and a stool kit to collect their stool in the morning of visit 2.

Subjects who met all of the study inclusion criteria and none of the exclusion criteria were assigned a randomization number at visit 2. Subjects were randomized into one of two groups based on an atmospheric method for randomization. One group was assigned the active product for visits 2, 3, and 4, followed by placebo for visits 5, 6, and 7. The other group was assigned placebo for visits 2, 3, and 4, followed by the active product for visits 5, 6, and 7. Study subjects and researchers were blinded as to which product contains the active product or placebo.

After a two-week washout period of any dietary supplements and lactose-containing products, subjects arrived in the clinic for their baseline visit (visit 2), where they were interviewed by the clinical staff to determine changes in their medical history and whether they started any new medications. Subjects also returned their completed diaries and used stool kits at this visit. Subjects who qualified were assigned a randomization number and performed the Lactose Challenge Test (HBT and 6-h SSC). A two-week supply of the study product along with standardized foods and stool kit were dispensed by the end of the visit. Subjects returned to the clinic at visit 3 (week 2) and visit 4 (week 4) for repeat assessments. After a two-week washout period, crossover of products was performed. Crossover of study product was performed at visit 5, where subjects who had received placebo at visit 2 would receive product at visit 5, and those who received product at visit 2 would receive placebo at visit 5. Otherwise, pre-visit preparation and visit procedures for visits 5, 6, and 7 were conducted in a manner identical to visits 2, 3, and 4. An outline of this visit breakdown is demonstrated in Table 2. The breakdown of HBT and symptom score measurement is outlined in Table 3.

**Table 2:** Visit schedule

Protocol Activity	V1 (Screening)	V1.5 (Screening)	Washout period	V2 (Arm 1)	V3 (Arm 1)	V4 (Arm 1)	Washout period	V5 (Arm 2)	V6 (Arm 2)	V7 (Arm 2)
	Week (−2)			Week 0	Week 2	Week 4		Week 0	Week 2	Week 4
Informed consent process	x									
Inclusion/Exclusion	x									
Medical history & physical exam	x									
Vital signs	x	x		x	x	x		x	x	x
Urine pregnancy test	x									
Standardized meals	x	x		x	x	x		x	x	
Administer & review scales and questionnaires				x	x	x		x	x	x
Review concomitant therapies		x		x	x	x		x	x	x
Intercurrent medical issues review		x		x	x	x		x	x	x
Randomization/study product preparation & dispensing				x	x			x	x	
Hydrogen breath test		x		x	x	x		x	x	x
Fecal collection				x	x	x		x	x	x

**Table 3:** 6-Hour symptom score and Hydrogen breath testing

	Pre-Lactose (−10 min)	1 hours	2 hours	3 hours	4 hours	5 hours	6 hours
6-Hour symptom score	X	X	X	X	X	X	X
Hydrogen breath testing	X	X	X	X

**Table 4:** Within-group analysis of placebo group-Week 4, Hour 0 to Hour 6

	Time point	*n*	Mean	Std. deviation	Std. error mean	*p*-value	Percent change
Diarrhea	Hour 0	24	0.42	.974	0.199	0.006 *	380.00 %
	Hour 6	24	2.00	2.519	0.514		
Abdominal cramping	Hour 0	24	1.04	1.517	0.310	0.004 *	124.00 %
	Hour 6	24	2.33	2.014	0.411		
Vomiting	Hour 0	24	0.21	0.415	0.085	1.000	80.00 %
	Hour 6	24	0.38	0.970	0.198		
Audible bowel sounds	Hour 0	24	1.54	1.693	0.346	0.053	48.65 %
	Hour 6	24	2.29	2.216	0.452		
Flatulence	Hour 0	24	1.58	1.840	0.376	0.167	71.05 %
	Hour 6	24	2.71	2.545	0.519		
Overall	Hour 0	24	4.79	5.610	1.145	0.017	102.61 %
	Hour 6	24	9.71	8.379	1.710		

*statistically significant at *p* < 0.01

**Table 5:** Within-group analysis of active group - Week 4, Hour 0 to Hour 6

	Time point	*n*	Mean	Std. deviation	Std. error mean	*p*-value	Percent change
Diarrhea	Hour 0	22	0.45	1.595	0.340	0.289	190.00 %
	Hour 6	22	1.32	2.255	0.481		
Abdominal cramping	Hour 0	22	.64	1.177	0.251	0.227	128.57 %
	Hour 6	22	1.45	2.087	0.445		
Vomiting	Hour 0	22	0.05	0.213	0.045	1.000	0.00 %
	Hour 6	22	0.05	0.213	0.045		
Audible bowel sounds	Hour 0	22	1.45	1.711	0.365	0.057	46.88 %
	Hour 6	22	2.14	1.959	0.418		
Flatulence	Hour 0	23	1.30	1.743	0.364	0.004 **	150.00 %
	Hour 6	23	3.26	2.562	0.534		
Overall	Hour 0	22	3.86	5.167	1.102	0.027 *	112.94 %
	Hour 6	22	8.23	7.502	1.599		

*statistically significant at *p* < 0.05; ** statistically significant at *p* > 0.01

**Table 6:** Linear mixed model of active vs. placebo at Week 4

	Time point	*n*	Mean	Std. Deviation	Std. Error Mean	*p*-value
Diarrhea	Active	147	1.34	2.462	0.203	0.033 *
	Placebo	147	1.69	2.558	0.211	
Abdominal cramping	Active	147	1.94	2.341	0.193	0.012 *
	Placebo	147	2.39	2.188	0.180	
Vomiting	Active	147	.08	.379	0.031	0.0002 **
	Placebo	147	.36	.936	0.077	
Audible bowel sounds	Active	147	2.76	2.536	0.209	0.589
	Placebo	147	2.86	2.497	0.206	
Flatulence	Active	148	3.16	2.873	0.236	0.770
	Placebo	148	3.21	2.699	0.222	
Overall	Active	147	9.28	9.202	0.759	.037 *
	Placebo	147	10.51	9.327	0.769	

This table describes all of the time points at week 4, comparing the active versus placebo groups

*statistically significant at *p* < 0.05; ** statistically significant at *p* > 0.01

### Endpoints

The purpose of this study was to determine the efficacy of the DDS-1 study product on providing symptomatic relief for lactose intolerance. The primary objective was to compare the effect of the study product to placebo on relieving lactose intolerance symptoms through changes in 6-h Symptom Scores from baseline following lactose challenge. Symptoms include diarrhea, abdominal cramping, vomiting, bowel sounds, flatulence, and overall symptoms. Severity of symptoms was evaluated using an 11-point scale from 0 (no symptoms) to 10 (most severe symptoms).

The secondary objective was to compare the effect of the DDS-1 study product to placebo on improving lactose digestion assessed by hydrogen breath test. Stool form was assessed using the Bristol Stool Scale. Daily GI questionnaires were also administered and included assessments of bowel urgency, abdominal bloating, abdominal discomfort, satisfaction with bowel habits, flatulence, burping, early satiety, nausea, vomiting, and borborygmi. The other objectives were to assess the levels of bacterial cultures in the stool and the effect of the study product on quality of life as measured by a quality of life questionnaire.

### Statistics

Parallel dual data entries were performed across all endpoints, followed by data validation and reconciliation of parallel entry. The monitoring team compared the values on the original CRFs or source documents, correcting any discrepancies found. All data elements were screened for reasonableness, and all missing, suspicious, or impossible values were referred back to the monitoring team for query generation and resolution. The database was formally locked after all flagged entries in the database were resolved. The locked database was free of any changes on the data sources and data entries. After closure of the study, the product assignments were then distinguished from the blinding codes and merged into a database for unblinding the data. The product assignments were then distinguished from the randomization or blinding codes and merged into the database and data tables.

A modified per protocol (Mod PP) analysis was performed to assess the efficacy variables of the study. Subjects with at least one post-dose visit completed were included in the analysis. Differences within time periods for each arm (within group test) and differences between two arms for each time point (between group tests) were analyzed for each endpoint. Descriptive measures such as means, standard deviations, and standard errors of means were processed for each numeric endpoint on all visits. Percentage changes were used to quantify increase or decrease of endpoints from baseline for each arm. Categorical endpoints were presented as frequency tables, with corresponding percentages. For each endpoint in ordinal scale, the differences in the medians within time periods for each arm were tested for nominal significance using non-parametric test (Wilcoxon Signed Rank Test or Sign Test). For each endpoint in the interval/ratio scale that followed semblance to normality, the difference from baseline to each subsequent time point was tested for each arm for nominal significance using the paired *t*-test. If the data was found to violate assumptions on normality, the non-parametric Wilcoxon Signed Ranks test or Sign Test was used. For continuous endpoints at each time point, the difference between means of different arms was assessed for significance using either the paired Student's *t*-test for endpoints satisfying parametric assumptions or the non-parametric Wilcoxon Signed Ranks test or Sign Test. For categorical endpoints, the Sign Test was used to compare the difference between arms for each time point.

All efficacy endpoints were analyzed depending on the level of measurement of the endpoint. The 6-h symptom score was analyzed using paired Student's *t*-test or by the non-parametric Wilcoxon Signed Ranks test or Sign Test for those data that were found to be substantially non-normally distributed. Significant changes in occurrence of bowel movements of related samples were observed weekly. In order to provide a longitudinal comparison for changes in symptoms between the active and placebo groups, a linear mixed model analysis was also performed.

Outcomes from the HBT were evaluated using the univariate general linear model. Since the study was a crossover study, three factors were considered: (1) the product applied before the endpoint was measured, (2) the order in which the product was applied, and (3) the randomization group.

To obtain comparable documentation on adverse events (AE), the investigator asked the subject the open, standardized, questions at each visit. Frequency and intensity of adverse events, including assessment of seriousness, were recorded in detail during each visit. Differences in AE patterns between product groups were assessed by McNemar Change Test.

## Results

**Fig. 1:**
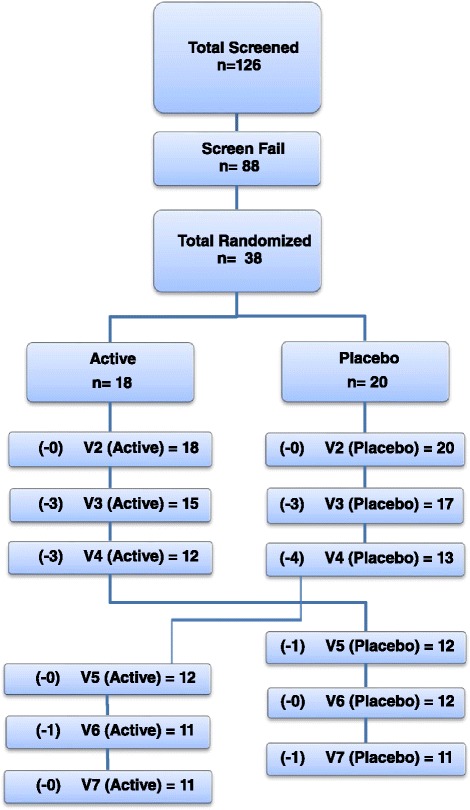
Attrition chart

Of the 126 individuals screened, 38 were randomized to receive the study product or placebo, of which 18 were initially placed in the product group and 20 in the placebo group. Fifteen subjects (7 in the active group and 9 in the placebo group) terminated the study early due to relocation, loss to follow-up, or voluntary subject withdrawal. Of these early termination subjects, one subject completed the active portion and two subjects completed the placebo portion of the study. No subject withdrawals were related to adverse events. A total of 22 subjects completed both crossover arms of the study and were included in the final analysis set, including 11 subjects initially assigned to the active group on visit 2 and 11 subjects initially assigned the placebo group on visit 2. For within-group analysis, the total sample sizes were 23 for the active group and 24 for the placebo group. Of note, one subject that completed only the active portion of the study provided reliable data only for the flatulence measure. Thus the total size of the active group was 22 for all measures except for flatulence, which was 23. A graphical representation of the attrition data is presented in Fig. 1.

Within-group analysis of the placebo group demonstrated that after four weeks, a statistically significant increase in symptoms of abdominal cramping (1.04 at hour 0 to 2.33 at hour 6, *p* = 0.004) and overall symptom score (4.79 at hour 0 to 9.71 at hour 6, *p* = 0.017) was noted (Table 4). During this period, the product group also demonstrated significant increases in overall symptom score (3.86 at hour 0 to 8.23 at hour 6, *p* = 0.027), as well as significantly increased flatulence (1.30 at hour 0 to 3.26 at hour 6, *p* = 0.027) (Table 5).

Using the linear mixed model, longitudinal comparison between the DDS-1 and placebo group demonstrated a statistically significant difference at week 4 in the diarrhea symptom score (*p* = 0.033; 1.34 in the active group compared to 1.69 in the placebo group), as well as abdominal cramping (*p* = 0.012; 1.94 compared to 2.39 in the placebo group), vomiting (*p* = 0.002; 0.08 compared to 0.36 in the placebo group), and overall (*p* = 0.037; 9.28 compared to 10.51 in the placebo group) (Table 6).

The 6-h symptom scores for Bowel Sounds and Flatulence were not statistically significantly different between the DDS-1 and placebo group at any time point during the course of the study. Furthermore, no statistically significant differences between groups were observed for the HBT, stool form as documented on Bristol Stool Scale, and the SF-12 quality of life survey by the end of the study. Although stool samples were collected, analysis of the stool was not completed due to budgetary limitations as well as positive findings based on 6-h symptom scores.

## Discussion

In the present study, the effect of the DDS-1 study product on relieving lactose intolerance symptoms was compared to placebo. This study aimed to include subjects reporting symptoms of lactose intolerance including diarrhea, abdominal cramping, vomiting, audible bowel sounds, and flatulence. Longitudinal evaluation found the group receiving the placebo to have worsened symptoms on lactose challenge at 4 weeks compared with the group receiving the DDS-1 strain of *Lactobacillus acidophilus*. Of note, data on HBT, stool form, and the SF-12 quality of life survey did not yield statistically significant results. Thus, for the purpose of brevity, these results were not reported.

Use of probiotics in management of lactose maldigestion stems from both anecdotal and empirical evidence related to cultured yogurt consumption. Cultured yogurt has been shown to possess considerable enzyme activity primarily due to the lactase produced by probiotic bacteria such as *Lactobacillus acidophilus*, *Lactobacillus bulgaricus* and *Streptococcus thermophilus* [24–27]. A clinical study on 10 lactase non-persistent individuals, ingestion of 18 g of lactose in yogurt resulted in significantly lower hydrogen excretion compared to ingestion of milk with an equal amount of lactose, implying a better absorption of lactose in yogurt. Fewer reports of diarrhea or flatulence were also observed in subjects who consumed yogurt [26]. The bacterial lactase is thought to be superior to synthetic lactase enzyme due to the ability to withstand the acidity of the stomach through encasement within the bacterial cells. As the yogurt enters the small intestine, the slower gastrointestinal transit time permits the activation of lactase, digesting lactose efficiently. Bile is then thought to emulsify the bacterial cell walls, resulting in release of lactase in the intestinal tract [27, 45]. Thus the enzyme activity in yogurt depends upon the buffer capacity of the microbial cells in yogurt to resist stomach acid as well as the effect of bile on the microbial cell to release beta-galactosidase. Pasteurization is also thought to reduce lactose auto-digestion capacity [27, 46–48].

Altering the microbial flora through consumption of probiotic supplements is thought to support improved lactose tolerance. Studies have yielded mixed results on the efficacy of probiotics in improving breath test results or improving abdominal symptoms [10, 20, 49, 50]. These mixed results may be related to variations in preparation of the probiotic product. This study uses a unique strain of *Lactobacillus acidophilus* known as DDS-1. This unique strain of *Lactobacillus acidophilus* was discovered by Dr. Khem Shahani at the University of Nebraska in 1959. A recent single-blind, crossover study of twelve subjects revealed that *Lactobacillus acidophilus* DDS-1 persisted in the gastrointestinal tract 1 day after consumption, indicating that the strain survived gastric passage [43]. After an 8-day washout, however, the probiotic strains were no longer detected. With the knowledge that DDS-1 persists in the gut, we aimed to determine for the first time in a randomized, double-blind, crossover design if the DDS-1 strain was more effective than placebo in alleviating symptoms of lactose intolerance.

The results of this study suggest that although no significant changes were observed for HBT, the DDS-1 strain of *Lactobacillus acidophilus* can help improve gastrointestinal symptoms of lactose intolerance such as diarrhea, abdominal cramping, and vomiting. The lack of significant effects on HBT further establishes that lactose malabsorption and lactose intolerance carry some degree of mutual exclusivity [49]. This is further demonstrated through a randomized trial on subjects with self-reported lactose intolerance that found ingestion of *Lactobacillus acidophilus* BG2FO4, a strain with high lactase activity and strong intestinal adherence as measured in an in vitro experiment, twice a day for seven days did not significantly alter overall hydrogen production compared to baseline [49]. This study also did not find significant changes in symptom scores. Based on this, the findings of our study may suggest that changes in HBT occur after improvement in symptoms.

Of note, subjects during visit 4 of the active phase were still noted to have increased symptoms of flatulence after 4 weeks, which was not increased in the placebo group. Many studies report improved flatulence after consumption of probiotics [20, 51, 52], thus this may represent spurious data related to insufficient statistical power. Alternatively, it has been suggested that initial use of *Lactobacillus* supplements may include abdominal discomfort and increased flatulence while the probiotic bacteria colonizes in the intestinal tract [53]. Thus a longer duration study may be employed to better understand the relationship between DDS-1 and flatulence.

This study provides preliminary evidence that the DDS-1 strain of *Lactobacillus acidophilus* not only persists in the gastrointestinal tract after prolonged use, but also can provide symptom benefit compared with placebo among individuals who consume the product for a course of 4 weeks. This study did not determine the number of patients with actual lactose maldigestion. It is possible, therefore, that not all patients who believe that they are lactose intolerant are actual maldigesters. Future randomized, placebo-controlled clinical trials are warranted to confirm the results of this study and to further determine the efficacy of the DDS-1 strain on other therapeutic areas which lactobacillus is postulated to affect, such as the immune system, cholesterol levels, microbial infection, and cancer.

### Limitations

A major limitation of this study is the limited time frame of four weeks. While many studies have demonstrated responses to probiotic consumption within this time frame, extension of both active and placebo groups may have demonstrated a more significant improvement in the 0-h to 6-h symptom score after prolonged use. This may be particularly true for the flatulence measurement, which was found to significantly increase on lactose challenge after 4 weeks of probiotic use. Prior studies have noted improvement in flatulence symptoms after probiotic use [20, 51, 52]. Similarly, lack of significant change in hydrogen breath testing may be related to insufficient time for the probiotic bacteria to fully colonize, as seen in other studies with treatment phases of 4 weeks or less [49, 54].

Another limitation of this study is the absence of stool sample data demonstrating persistence of lactobacillus strain in the stool. However, it has been previously reported that the DDS-1 strain does successfully establish in the human gastrointestinal tract [43].

## Conclusion

The present study has found that this unique DDS-1 strain of *Lactobacillus acidophilus*, manufactured by Nebraska Cultures, Inc., is safe to consume and improves abdominal symptom scores with respect to diarrhea, cramping, and vomiting during an acute lactose challenge.

## Acknowledgement of guidance on competing interests

BioMed Central's guidance on competing interests have been reviewed in detail. Of note, ethics approval has been obtained by an independent institutional review board (IRB). Additionally, a detailed consent process with documentation of signed consent is completed prior to any study-related procedures.

Medicus Research is a third-party contract research organization that was funded by Nebraska Cultures to perform this study. No member of Nebraska Cultures was involved in the study design, execution, or interpretation of study data. The only role of Nebraska Cultures in this study was funding of the study itself and providing the DDS-1 study product. Michael Shahani, the COO of Nebraska Cultures, was involved in describing the product preparation in the methods section of the manuscript, however played no role in any of the remaining portions of the manuscript, such as statistical analysis or discussion.

## Disclosures of conflict of interest by Author and Co-Authors

Jay K. Udani was employed by Medicus Research. Medicus Research received research funding for this study from Nebraska Cultures. MS is an employee of Nebraska Cultures, Inc. MNP and JPM were independently hired to draft the results of this study. MNP, JKU, JPM, Pakdaman Consulting, and Medicus Research do not endorse any brand or product. Medicus Research and Pakdaman Consulting do not have any financial interests with any supplement manufacturer or distributor.

## Sources of financial support

This study was conducted independently by JKU, under the sponsorship of Nebraska Cultures, Inc. Nebraska Cultures was not involved in the preparation or conduction of the clinical trial.

MNP and JPM are affiliated with Pakdaman Consulting, Inc., an independent research organization that was funded by Nebraska Cultures for preparation of this manuscript. MS is affiliated with Nebraska Cultures. Mr. Shahani's involvement in the manuscript involved description of the study product preparation. Mr. Shahani was not involved in data analysis or discussion.

## Consent to publish

An important component of the consent documentation includes consent from the participant (or legal parent or guardian for children) to report individual patient data. However, while all participants were consented for this, no individual patient data is reported in this manuscript.
