# Erratum to: the effects of the DDS-1 strain of lactobacillus on symptomatic relief for lactose intolerance - a randomized, double-blind, placebo-controlled, crossover clinical trial

**DOI:** 10.1186/s12937-016-0199-0

**Published:** 2016-10-03

**Authors:** Michael N. Pakdaman, Jay K. Udani, Jhanna Pamela Molina, Michael Shahani

**Affiliations:** 1 Pakdaman Consulting, Inc., 22287 Mulholland Hwy, Calabasas, CA 91302 USA; 2 Northridge Hospital Integrative Medicine Program, 18300 Roscoe Blvd, Northridge, CA 91328 USA; 3 Nebraska Cultures, 45 Quail Ct #206, Walnut Creek, CA 94596 USA

## Erratum

This erratum is in response to comments made to our recently published study “The Effects of the DDS-1 Strain of Lactobacillus on Symptomatic Relief for Lactose Intolerance - A Randomized, Double-Blind, Placebo-Controlled, Crossover Clinical Trial” [1].

The Frese et. al. study [2] compares autochthonous strains of Lactobacillus to DDS-1, an allochthonous strain. This is a significant aspect of their study, as an allochthonous strain would generally not be expected to establish in higher levels compared with a strain naturally found in the human gastrointestinal tract. The comment “the DDS-1 strain of *L. acidophilus* to be superior to other strains of lactobacillus in the ability to establish in the human gastrointestinal (GI) tract [2]” refers to its ability to colonize the GI tract compared with other allochthonous strains, which in other studies have not been shown to establish well in the human gastrointestinal tract. While it is true what the reader states that “authors found that only in a small number of subjects did the strain survive gastric passage, and the strain did so in lower numbers and in fewer subjects than the other Lactobacillus species tested” - this is expected as an allochthonous strain (eg DDS-1) would not be expected to demonstrate superior colonization compared with a strain naturally found in the human gastrointestinal tract (e.g. Lactobacillus reuteri and mucosae).

With regard to the statement “However, it has been previously reported that the DDS-1 strain does successfully establish in the human gastrointestinal tract [2].” This statement is true. It is important to note that nowhere in the article do we suggest that someone consume DDS-1 for 8 days, then discontinue the product altogether and expected for it to work. The purpose of our reference to that study is that DDS-1 does successfully establish in the gastrointestinal tract. We made no comments on for how long or if it is superior than strains naturally occurring in the human body.

With regard to the reader’s comment “It is clear from this previous work that the allochthonous DDS-1 is a poor choice with respect to gastric transit, establishment of Lactobacillus populations in the gastrointestinal tract, and in comparison to other Lactobacillus species,” we disagree. While we agree that it is not superior to strains naturally found in the human gastrointestinal tract (eg Lactobacillus reuteri and mucosae), it is not expected to be so. The purpose for reference to the Frese et al. article was to demonstrate that the DDS-1 strain of *L. Acidophilus* in fact does colonize, suggesting that symptom improvement during consumption can be potentially linked to this colonization.
